# Multiple imputation for discrete data: Evaluation of the joint latent normal model

**DOI:** 10.1002/bimj.201800222

**Published:** 2019-03-14

**Authors:** Matteo Quartagno, James R. Carpenter

**Affiliations:** ^1^ Department of Medical Statistics London School of Hygiene and Tropical Medicine London UK; ^2^ MRC Clinical Trials Unit at UCL 90 High Holborn London UK

**Keywords:** categorical data, joint model, latent normal model, missing data, multiple imputation

## Abstract

Missing data are ubiquitous in clinical and social research, and multiple imputation (MI) is increasingly the methodology of choice for practitioners. Two principal strategies for imputation have been proposed in the literature: joint modelling multiple imputation (JM‐MI) and full conditional specification multiple imputation (FCS‐MI). While JM‐MI is arguably a preferable approach, because it involves specification of an explicit imputation model, FCS‐MI is pragmatically appealing, because of its flexibility in handling different types of variables. JM‐MI has developed from the multivariate normal model, and latent normal variables have been proposed as a natural way to extend this model to handle categorical variables. In this article, we evaluate the latent normal model through an extensive simulation study and an application on data from the German Breast Cancer Study Group, comparing the results with FCS‐MI. We divide our investigation in four sections, focusing on (i) binary, (ii) categorical, (iii) ordinal, and (iv) count data. Using data simulated from both the latent normal model and the general location model, we find that in all but one extreme general location model setting JM‐MI works very well, and sometimes outperforms FCS‐MI. We conclude the latent normal model, implemented in the R package jomo, can be used with confidence by researchers, both for single and multilevel multiple imputation.

## 1 INTRODUCTION

Over recent years, the popularity of multiple imputation (MI) as a tool for the analysis of clinical and social data with missing observations has continued to increase. Restricting analysis to complete records (CR) excludes information from all units with one or more missing values, and may be prone to bias if data are missing at random (MAR) and missingness is dependent on the outcome of the analysis model. By contrast, MI uses all the available data, is efficient, and gives valid inference if data are MAR.

Multiple imputation proceeds by first forming the Bayesian predictive distribution of the missing data, given the observed data. This is used to impute multiple “complete” datasets, which between them properly reflect the loss of information due to missing data. Then, the substantive scientific model is fitted to each imputed dataset in turn, and the results are combined for inference using Rubin's multiple imputation rules. For a detailed discussion and justification of the approach, see, for example, Carpenter and Kenward (2013), ch. 2.

As discussed in Carpenter and Kenward (2013) ch. 3–5, there are a number of different ways in which we can impute the missing data. In most situations, data are missing in multiple variables, i.e. in both outcome and covariates in the substantive scientific model. In these cases, there are two main strategies for imputing the missing values: full conditional specification multiple imputation (FCS‐MI) and joint modelling multiple imputation (JM‐MI).

FCS‐MI (van Buuren, Brand, Groothuis‐Oudshoorn, & Rubin, 2006), also known as imputation by chained equations (ICE), builds on the idea of imputing the missing values for each partially observed variable by setting up a univariate model fully conditional on all the other variables. By contrast, JM uses a joint multivariate model for all the partially observed data. Once the joint distribution has been defined, a Gibbs sampler is used to update the parameters of this model and the missing data are imputed from the proper conditional distribution given the observed data and the current values of the parameters.

As long as the joint imputation model is (reasonably) compatible with the analysis model, JM imputation is the theoretically best way of imputing the data and it leads to unbiased inference under MAR. When FCS is used, the joint model for the data is only defined implicitly. Care needs to be taken to ensure the various conditional specifications are not inconsistent. Further, establishing conditions for the validity of the method is more difficult, although the method has been shown to perform well in practice. However, recently, conditions for equivalence of FCS to JM have been explored in two different studies (Hughes et al., 2014; Liu, Gelman, Hill, Su, & Kropko, 2013), which broadly concluded that:

- the two methods are equivalent when the joint model is multivariate normal;
- the two methods are equivalent when the so‐called “noninformative margins” condition holds, otherwise an order effect occurs, and
- in real data the magnitude of this order effect is usually small enough to be considered negligible.

Therefore the two methods seem to be interchangeable in most situations. However, FCS has become more popular than JM and the main reasons for its success are:

- its relative flexibility for accommodating different kinds of variables; e.g. linear regression can be used for the univariate conditional model to impute continuous variables, while a logistic model can be used for binary data, a Poisson for count data, *etc*., and
- the availability of well‐maintained and accessible software packages, for example ICE (now MI impute chained, Stata) and *mice* (R, van Buuren and Groothuis‐Oudshoorn (2011)).

Finding an appropriate joint model with noncontinuous variables, for example binary or categorical variables, is more challenging. Lee and Mitra (2016) proposed a method based on sequential generalized linear models, while Schafer (1997) proposed a strategy based on a general location model. On the software front, some good packages for JM‐MI are available, but to the best of our knowledge none of them is able to handle a mix of partially observed continuous and categorical variables in a way that naturally extends to a multilevel setting. One approach that has been suggested is to simply treat binary variables as continuous in the imputation model (see the discussion in Carpenter & Kenward, 2013, ch. 4–5). This can be a reasonable approach when the partially observed binary variables are included as covariates in the substantive analysis model. However, a proportion of the imputed values will fall outside of the 0/1 set of values. Rounding can be used, but its use has been discouraged and shown to be prone to bias in certain settings (Horton, Lipsitz, & Parzen, 2003).

A more theoretically sound approach is to make use of latent normal variables to handle noncontinuous data, as suggested in Goldstein, Carpenter, Kenward, and Levin (2009). We developed the R package *jomo* (Quartagno & Carpenter, 2014), which allows for the imputation of a mix of continuous, binary or nominal data under this model. The aim of this paper is to explore, via a comprehensive set of simulations, the validity and practical utility of the latent normal model for imputing a mix of partially observed continuous and categorical data.

The article is structured as follows. In Section 2, we describe the two approaches we compare, namely imputation by joint modeling and full conditional specification. In Section 3, we report the results of a series of increasingly challenging simulation studies, designed to establish the validity of the latent normal approach to categorical variables in increasingly complex models. Section 4 illustrates the approaches using data derived from a cohort study from the German Breast Cancer Study Group. We conclude with a discussion in Section 5. All the simulations reported here were carried out with the freely available R‐package jomo (Quartagno, Grund, & Carpenter, 2018).

## 2 METHODS

We now give an overview of Joint Modelling Multiple Imputation (JM‐MI), presenting the general form of the imputation model which we evaluate in the subsequent simulation studies.

Suppose we intend to collect *N* observations on *K* variables $Y_{k}$, but we end up with missing values in each (or at least some) of these variables. To use JM‐MI to impute these missing data, the first step is to set up the joint model for the partially observed data. If the data are plausibly multivariate normal, we use the multivariate normal model:

(1) $$\left\{\begin{array}{c} y_{i,1}=\beta_{0,1}+\varepsilon_{i,1}\\ \vdots \\ y_{i,k}=\beta_{0,k}+\varepsilon_{i,k}\\ \vdots \\ y_{i,K}=\beta_{0,K}+\varepsilon_{i,K} \end{array}\right.,\;\left(\begin{array}{c} \varepsilon_{i,1}\\ \vdots \\ \varepsilon_{i,k}\\ \vdots \\ \varepsilon_{i,K} \end{array}\right)∼N\left[\left(\begin{array}{c} 0\\ \ldots \\ 0\\ \ldots \\ 0 \end{array}\right),\Omega_{e}\right],$$

where β coefficients are the fixed effect parameters, ε the error terms and $\Omega_{e}$ is the unstructured variance‐covariance matrix of the residuals. When some of the *K* variables are binary or categorical, Goldstein et al. (2009) proposed a natural extension via latent normal variables, based on a previous proposal by Albert and Chib (1993). To understand the approach, suppose that $Y_{i,k}$ is a binary variable, and that we include in the model not $Y_{i,k}$, but instead two latent normal variables, one for each level of $Y_{i,k}$. Denote these two normal variables by $Z_{i,1,k}$ and $Z_{i,2,k}$, where $Y_{i,k}=1$ if $Z_{i,2,k}>Z_{i,1,k}$ and 0 otherwise. Model (1) becomes:

(2) $$\left\{\begin{array}{c} y_{i,1}=\beta_{0,1}+\varepsilon_{i,1}\\ \vdots \\ z_{i,k,1}=\beta_{0,k,1}+\varepsilon_{i,k,1}\\ z_{i,k,2}=\beta_{0,k,2}+\varepsilon_{i,k,2}\\ \vdots \\ y_{i,K}=\beta_{0,K}+\varepsilon_{i,K} \end{array}\right.\;\left(\begin{array}{c} \varepsilon_{i,1}\\ \ldots \\ \varepsilon_{i,k,1}\\ \varepsilon_{i,k,2}\\ \vdots \\ \varepsilon_{i,K} \end{array}\right)∼N\left[\left(\begin{array}{c} 0\\ \vdots \\ 0\\ \ldots \\ 0 \end{array}\right),\Omega_{e}\right].$$

Unfortunately, this model is nonidentifiable (because we have replaced a binary variable, with one parameter, by two latent normals whose joint distribution has five parameters). However, this can be addressed with two simple tweaks: (i) we fix the value of the variance for the latent normals to an arbitrary value, say for example to 0.5, and (ii) we subtract the equation for $Z_{i,2,k}$ from the one for $Z_{i,1,k}$:

(3) $$\left\{\begin{array}{c} y_{i,1}=\beta_{0,1}+\varepsilon_{i,1}\\ \vdots \\ z_{i,k}=\beta_{0,k}+\varepsilon_{i,k}\\ \vdots \\ y_{i,K}=\beta_{0,K}+\varepsilon_{i,K} \end{array}\right.\;\left(\begin{array}{c} \varepsilon_{i,1}\\ \vdots \\ \varepsilon_{i,k}\\ \vdots \\ \varepsilon_{i,K} \end{array}\right)∼N\left[\left(\begin{array}{c} 0\\ \vdots \\ 0\\ \vdots \\ 0 \end{array}\right),\Omega_{e}\right]\;\omega_{e,k,k}^{2}=1,$$

where the variance of the remaining latent normal is now 1, because it is obtained from the subtraction of two normals with variance 0.5. In this new formulation, it follows that binary $Y_{i,k}=1$ if $Z_{i,k}>0$ and 0 otherwise.

The same reasoning extends naturally to *T*‐level unordered categorical variables. In the resulting model, we have T−1 latent normals, each of which has a fixed variance of 1, and covariance with the other latent normal variables of 0.5. For example, in a situation where we have a continuous variable, a 4‐level categorical variable and a binary variable, the latent normal model is:

(4) $$\begin{array}{c} y_{i,1}=\beta_{0,1}+\varepsilon_{i,1}\\ z_{i,2,1}=\beta_{0,2,1}+\varepsilon_{i,2,1}\\ z_{i,2,2}=\beta_{0,2,2}+\varepsilon_{i,2,2}\\ z_{i,2,3}=\beta_{0,2,3}+\varepsilon_{i,2,3}\\ z_{i,3}=\beta_{0,3}+\varepsilon_{i,3}\\ \left(\begin{array}{c} \varepsilon_{i,1}\\ \varepsilon_{i,2,1}\\ \varepsilon_{i,2,2}\\ \varepsilon_{i,2,3}\\ \varepsilon_{i,3} \end{array}\right)∼N\left[\left(\begin{array}{c} 0\\ 0\\ 0\\ 0\\ 0 \end{array}\right),\Omega_{e}=\left(\begin{array}{ccccc} \omega_{1,1}^{2} & \omega_{1,2} & \omega_{1,3} & \omega_{1,4} & \omega_{1,5}\\ \omega_{2,1} & 1 & 0.5 & 0.5 & \omega_{2,5}\\ \omega_{3,1} & 0.5 & 1 & 0.5 & \omega_{3,5}\\ \omega_{4,1} & 0.5 & 0.5 & 1 & \omega_{4,5}\\ \omega_{5,1} & \omega_{5,2} & \omega_{5,3} & \omega_{5,4} & 1 \end{array}\right)\right]. \end{array}$$

This model has two sets of parameters: the fixed effect parameters $\beta =(\beta_{0,1},\beta_{0,2,1},\beta_{0,2,2},\beta_{0,2,3},\beta_{0,3})$ and the covariance matrix $\Omega_{e}$. Fitting this model in the Bayesian framework using MCMC allows imputation of any missing values of the variables under the Missing At Random (MAR) assumption using the data‐augmentation approach (Tanner & Wong, 1987). We:

1. draw values of the parameters, given the data and the priors, using MCMC (Gibbs sampling where possible), then
2. draw the missing values given the observed data and current parameter draws.

As usual with MI, we run (update) this algorithm until it has stochastically converged, then keep the current draws of the missing values. Together with the observed data these make the first imputed dataset. Then, after a further set of updates, the current draws of the missing values are retained with the observed data to make the second imputed dataset, and so on. We choose the number of updates so that, conditional on the observed data, draws of the missing data in successive imputed datasets are independent.

We use uninformative priors (flat priors for the fixed parameters, β) to give the greatest weight to the data. At each step of the algorithm, we need to know the proper conditional distributions from which to draw the new parameter values. Unfortunately, because of the constraints in the covariance matrix, we cannot draw a new value for the covariance matrix from a known distribution. Therefore, we rely on a Metropolis–Hastings step to update it element‐wise, following Browne (2006).

Additionally, with the latent normal model, at each step we need to draw new values for the latent normal variables, $z_{i,2,}$ and $z_{i,3}$. This is done using a rejection sampling step: for each categorical variable in turn, for each individual, proposed values of the associated latent normal variables are drawn from the corresponding conditional normal distribution given the other observed data and draws of the missing data (or associated latent normals). This is repeated till the draws of the latent normals are consistent with the observed categorical value. For example, in the model above, if the 4‐level categorical variable $Y_{i,2}=4$, we draw the latent normal triple $\left(z_{i,2,1},z_{i,2,2},z_{i,2,3}\right)$ from the conditional normal distribution given $\left(y_{i,1},z_{i,3}\right)$ (at the current parameter values) until all three of $\left(z_{i,2,1},z_{i,2,2},z_{i,2,3}\right)$ are less than zero; we then accept this draw and move on. A detailed explanation and worked example is given in Carpenter and Kenward (2013, §5.2).

The algorithm presented in this section was developed to handle unordered categorical variables. A similar algorithm, but based on a single latent normal variable with appropriate thresholds between different categories, is again described in Goldstein et al. (2009) and it is implemented for example in softwares REALCOM (Carpenter, Goldstein, & Kenward, 2011) and M‐Plus (Muthen, Muthén, & Angeles, 2017). This should be more efficient when dealing with ordinal data, as it explicitly reflects the order between categories. However, it is not implemented in the R package *jomo*. One of the goals of this paper is to establish whether the use of the general algorithm for the imputation of unordered data when imputing ordinal variables is suitable, or whether the loss in efficiency is large.

## 3 SIMULATION STUDY

In this section, we present the results of a series of simulation studies designed to evaluate the use of latent‐normal JM‐MI with a mix of partially observed binary, categorical, ordinal, count, and continuous variables. We begin in Subsection 3.1 with a simple data generating model that matches (4). Then, we investigate what happens with different data generating mechanisms: in Subsection 3.2 we consider binary data, in Subsection 3.3 we consider categorical and ordinal data and lastly in Subsection 3.4 we consider count data.

Across all of the simulation scenarios, we present the results of the analyses with different methods in terms of mean estimates, mean standard error estimated from the models, empirical standard error (i.e. standard deviation of the simulation estimates) and coverage level. A valid method should yield unbiased results, similar model, and empirical standard errors and coverage levels close to 95%.

### 3.1 Matching latent normal model for data generation and imputation

Suppose we intend to collect i=1,⋯,N=1000 observations on three variables, continuous $y_{i}$, 4‐level categorical $x_{i,1}$ and binary $x_{i,2}$. The data generating model is:

(5) $$\begin{array}{c} \left(\begin{array}{c} y_{i,1}\\ z_{i,1,1}\\ z_{i,1,2}\\ z_{i,1,3}\\ v_{i,2} \end{array}\right)∼N\left[\left(\begin{array}{c} 2\\ -0.2\\ 0.6\\ 0\\ -0.1 \end{array}\right),\left(\begin{array}{ccccc} 2 & 0.5 & 0.5 & 0.5 & 0.5\\ 0.5 & 1 & 0.5 & 0.5 & 0.5\\ 0.5 & 0.5 & 1 & 0.5 & 0.5\\ 0.5 & 0.5 & 0.5 & 1 & 0.5\\ 0.5 & 0.5 & 0.5 & 0.5 & 1 \end{array}\right)\right], \end{array}$$

where $\left(z_{i,1,1},z_{i,1,2},z_{i,1,3}\right)$ is the latent normal triple corresponding to the 4‐level categorical variable $x_{i,1}$, and $v_{i,2}$ is the latent normal corresponding to binary $x_{i,2}$.

Next, suppose that the substantive analysis model is the following linear model:

(6) $$y_{i}=\beta_{0}+\beta_{1}I\left[x_{i,1}=2\right]+\beta_{2}I\left[x_{i,1}=3\right]+\beta_{3}I\left[x_{i,1}=4\right]+\beta_{4}x_{i,2}+\varepsilon_{i},$$

where I[.] is an indicator for the event in brackets.

We (i) simulate data from (5); (ii) make some values missing; (iii) impute using (4) and fit the substantive model (6) to the imputed data and (iv) combine the results for inference using Rubin's rules.

Missing values are generated as follows. Values of $y_{i}$ are made missing completely at random (MCAR) with probability 0.2. Independently, values of $x_{i,2}$ are made MCAR with probability 0.2. For $x_{i,1}$ we explore two different missingness mechanisms:

1. MCAR with probability 0.4;
2. for individuals *i* with $y_{i}$ observed, the probability of $x_{i,1}$ being missing is given by: ${\left(1+\exp\left(3-y_{i}\right)\right)}^{-1}$, leading to around 35% of $x_{i,1}$ MAR given *Y*.

Mechanism (1) is chosen to explore the extent to which JM‐MI can recover information relative to a complete records analysis, given that both methods lead to valid inference under MCAR. The second mechanism involves the outcome, and hence CR estimates are expected to lead to bias; we explore whether JM‐MI can successfully remove this bias.

The simulation study uses 1000 replications. For each replication, after generating the data and making values missing, we apply JM‐MI using the R package *jomo* (Quartagno & Carpenter, 2014), imputation model (4), a burn in of 500 updates and between‐imputation updates of 500 to generate 20 imputed datasets. Then we fit the substantive model (6) to (a) the remaining complete records after making data missing, and to (b) each of the 20 imputed datasets, combining the results using Rubin's rules.

For comparison, we also use standard FCS‐MI through the R package *mice*, using a linear regression model for the continuous variable, a logistic model for the binary variable and a multinomial logistic model for the categorical variable.

#### 3.1.1 Results

Table 1 shows the results. When data are MCAR, complete records analysis is valid and therefore, as expected, it gives consistent estimates of the parameters. However, the SEs show that MI recovers some information. Results are similar when using either JM‐MI or FCS‐MI, although with FCS there is a suggestion of undercoverage for β_1_ and β_3_.

**Table 1:** Results for subsection 3.1

	β_0_				β_1_				β_2_				β_3_				β_4_			
	Mean	mSE	eSE	Cov	Mean	mSE	eSE	Cov	Mean	mSE	eSE	Cov	Mean	mSE	eSE	Cov	Mean	mSE	eSE	Cov
True value	2.51			0.95	−0.05			0.95	−0.02			0.95	−0.79			0.95	−0.63			0.95
Full data	2.51	0.24	0.25	0.94	−0.05	0.25	0.26	0.95	−0.02	0.29	0.30	0.95	−0.79	0.30	0.30	0.95	−0.62	0.16	0.16	0.95
MCAR data:																				
Complete records	2.49	0.39	0.39	0.95	−0.04	0.41	0.41	0.95	−0.03	0.48	0.48	0.95	−0.79	0.49	0.49	0.95	−0.62	0.26	0.27	0.94
FCS‐MI	2.50	0.32	0.34	0.93	−0.04	0.35	0.38	0.91	−0.02	0.40	0.43	0.93	−0.79	0.41	0.44	0.92	−0.62	0.21	0.21	0.94
JM‐MI	2.50	0.34	0.34	0.95	−0.04	0.36	0.37	0.94	−0.02	0.43	0.42	0.95	−0.77	0.43	0.43	0.95	−0.63	0.21	0.21	0.95
MAR data:																				
Complete records	1.97	0.35	0.35	0.62	−0.05	0.36	0.37	0.95	−0.02	0.42	0.43	0.95	−0.64	0.41	0.43	0.91	−0.48	0.23	0.23	0.90
FCS‐MI	2.51	0.33	0.37	0.92	−0.07	0.36	0.41	0.90	−0.02	0.42	0.48	0.91	−0.76	0.41	0.45	0.92	−0.63	0.21	0.21	0.94
JM‐MI	2.49	0.35	0.35	0.95	−0.04	0.38	0.38	0.95	−0.02	0.45	0.44	0.94	−0.73	0.44	0.43	0.95	−0.64	0.21	0.21	0.95

Data are generated from (5), and made missing using the MCAR and MAR mechanisms described in the text. Mean, model, and empirical SE and coverage level are reported for the five model parameters in (6).

In the MAR scenario, the complete records estimates of β_0_, β_3_, and β_4_ are biased, as expected when data are MAR depending on the outcome of the analysis model. On the other hand, MI is valid under MAR, and therefore estimates should be unbiased. We see that JM‐MI gives good results for all parameters; however for FCS the MI standard error (mSE) is slightly underestimated for β_0_–β_3_, and in consequence the confidence interval coverage is slightly reduced.

### 3.2 Binary data

In the previous subsection, we generated data from a multivariate normal distribution, with latent normals used to determine values of the categorical and binary variables. From now on, we explore the performance of latent normal JM‐MI (with its implicit probit link functions for categorical variables) when data come from different data generating models, to give some insight into how the approach will perform in actual applications. We begin with binary data.

#### 3.2.1 Logistic regression

We generate data from a logistic model:

(7) $$\begin{array}{c} X_{1},X_{2}∼N\left(0,1\right)\\ Z=\beta_{0}+\beta_{1}X_{1}+\beta_{2}X_{2}\\ p=\frac{1}{1+exp(-Z)}\\ Y∼Bernoulli(p). \end{array}$$

Using a logistic link means this model is not strictly equivalent to the latent normal JM‐MI, which implicitly uses a probit link, although the differences between the two are small.

We generate data with three different sets of parameter values:

1. weak effects: β=(0.1,0.1,0.1);
2. moderate effects: β=(0.3,0.3,0.3), and
3. large effects: β=(3,3,3).

In all three scenarios, data are made missing independently in all three variables with probability ∼0.2, so that about 0.8^3^ = 0.51 of the records are complete. In particular, for *Y* and *X*_2_ we consider an MCAR mechanism, while for *X*_1_ we assume an MAR mechanism dependent on the outcome; this invalidates the complete records estimates.

We generate 1000 replications, each with N=300 observations, and compare CR, JM‐MI (imputation model with all variables as outcomes, i.e. similar to (4), same burn in and between imputation updates as before) and FCS‐MI (using a logistic model to impute the outcome and linear regression models to impute the covariates). We create 20 imputed datasets for each of the two imputation methods.

#### 3.2.2 Results

From Table 2, we can see that, in the small and medium effect scenarios, the estimates of the fixed effects after imputation are almost perfect, with negligible bias (<1%) and standard errors always smaller than in complete records analysis, irrespective of the imputation method.

**Table 2:** Results from data generating mechanism (7) and (9), with small, medium or large effects

	β_0_				β_1_				β_2_			
	Mean	mSE	eSE	Cov	Mean	mSE	eSE	Cov	Mean	mSE	eSE	Cov
Logit – small effects:												
True value	0.10			0.95	0.10			0.95	0.10			0.95
Full data	0.10	0.12	0.12	0.94	0.10	0.12	0.12	0.94	0.10	0.12	0.12	0.96
Complete records	−0.09	0.16	0.17	0.79	0.10	0.17	0.18	0.95	0.10	0.17	0.17	0.96
FCS‐MI	0.10	0.13	0.14	0.94	0.11	0.15	0.15	0.95	0.10	0.15	0.15	0.96
JM‐MI	0.10	0.13	0.14	0.94	0.10	0.15	0.15	0.96	0.10	0.15	0.15	0.95
True value	0.30			0.95	0.30			0.95	0.30			0.95
Full data	0.30	0.12	0.13	0.94	0.31	0.12	0.12	0.95	0.30	0.12	0.13	0.95
Complete records	0.11	0.17	0.17	0.80	0.32	0.17	0.18	0.95	0.30	0.17	0.19	0.94
FCS‐MI	0.30	0.14	0.14	0.94	0.32	0.16	0.16	0.95	0.30	0.16	0.16	0.94
JM‐MI	0.30	0.14	0.14	0.94	0.31	0.16	0.16	0.95	0.30	0.16	0.16	0.94
Logit – large effects:												
True value	3.00			0.95	3.00			0.95	3.00			0.95
Full data	3.12	0.43	0.45	0.95	3.13	0.46	0.47	0.96	3.13	0.46	0.50	0.95
Complete records	3.06	0.63	0.74	0.94	3.28	0.69	0.82	0.96	3.28	0.68	0.80	0.95
FCS‐MI	2.68	0.49	0.38	0.88	2.64	0.54	0.42	0.87	2.63	0.54	0.43	0.87
JM‐MI	3.12	0.58	0.65	0.95	3.11	0.64	0.72	0.94	3.11	0.63	0.72	0.94
Binary – small effects:												
True value	0.10			0.95	0.10			0.95	0.10			0.95
Full data	0.10	0.08	0.08	0.95	0.10	0.12	0.11	0.95	0.10	0.06	0.06	0.95
Complete records	−0.05	0.11	0.10	0.71	0.09	0.15	0.15	0.95	0.09	0.08	0.08	0.95
FCS‐MI	0.10	0.10	0.10	0.95	0.10	0.15	0.15	0.94	0.10	0.07	0.08	0.95
JM‐MI	0.10	0.10	0.10	0.95	0.10	0.15	0.15	0.94	0.10	0.07	0.08	0.94
Binary – medium effects:												
True value	0.30			0.95	0.30			0.95	0.30			0.95
Full data	0.30	0.08	0.08	0.96	0.30	0.12	0.11	0.95	0.30	0.06	0.06	0.94
Complete records	0.13	0.11	0.11	0.66	0.26	0.15	0.15	0.94	0.26	0.08	0.08	0.91
FCS‐MI	0.30	0.10	0.10	0.96	0.29	0.16	0.15	0.95	0.30	0.07	0.08	0.93
JM‐MI	0.30	0.10	0.10	0.95	0.29	0.16	0.15	0.95	0.30	0.07	0.08	0.93
Binary – large effects:												
True value	3.00			0.95	3.00			0.95	3.00			0.95
Full data	3.00	0.08	0.08	0.95	3.00	0.12	0.12	0.94	3.00	0.06	0.06	0.96
Complete records	2.90	0.11	0.11	0.85	2.85	0.17	0.17	0.86	2.86	0.10	0.10	0.72
FCS‐MI	3.02	0.11	0.11	0.94	2.99	0.15	0.15	0.95	3.02	0.09	0.08	0.97
JM‐MI	3.07	0.13	0.12	0.92	2.95	0.17	0.16	0.95	3.03	0.10	0.10	0.95

Mean, model, and empirical SE and coverage level are reported for all three model parameters. Data are MAR and we compare complete records (CR) to both FCS and JM multiple imputation.

**Figure 1:**
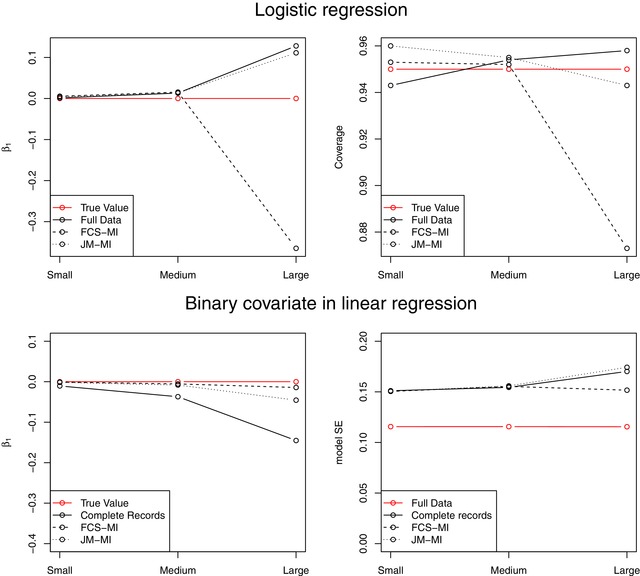
Results for binary data. Top panels: absolute bias (left) and coverage level (right) in the estimation of β_1_ in the logistic model (7) scenarios. Bottom panels: absolute bias (left) and model SE (right) in the estimation of β_1_ in the binary covariate (9) scenarios

When the effects are larger, even the full data estimates suffer from some small bias (as is typical with logistic regression). However, JM‐MI gives similar results to the full data and recovers information compared to CR, although the MI standard errors (mSE) are slightly smaller than the empirical standard errors (eSE). However, FCS‐MI suffers from bias that causes the coverage level to fall short of the 95% nominal level, possibly because the noninformative margin condition does not hold here. Absolute bias and coverage levels with the different methods are compared in the top panels of Figure 1.

#### 3.2.3 Binary covariate of regression model

Another situation where we need to impute a mix of binary and normal data is when we have some partially observed binary variables we wish to include as covariates in a linear regression. For example, suppose we observed continuous variables *Y* and *X*_2_ and binary *X*_1_ and that the substantive analysis model is:

(8) $$Y=\beta_{0}+\beta_{1}X_{1,i}+\beta_{2}X_{2,i}+\varepsilon_{i},\;\;\varepsilon_{i}\overset{iid}{∼}N\left(0,\sigma^{2}\right),$$

and that, consistent with (8) the data generation process is:

(9) $$\begin{array}{c} X_{1}∼\text{Bernoulli}\left(0.5\right)\;X_{2}∼N\left(0,1\right)\\ Y∼N(\beta_{0}+\beta_{1}X_{1}+\beta_{2}X_{2},1). \end{array}$$

As before, we simulate 1000 datasets with 300 observations each, and then independently generate missing data in each of the three variables with probability ∼0.2 (MCAR for Y and *X*_2_, MAR given the outcome for *X*_1_). We create 20 imputations with JM‐MI, with a model similar to (4), with all the variables as outcomes, and using FCS‐MI. Again, we explore three different scenarios with increasing effect magnitude.

#### 3.2.4 Results

The results are summarized in the bottom half of Table 2 and Figure 1. Again, in the two examples with smaller effect sizes, both MI methods behave well, giving unbiased fixed effect estimates and standard errors smaller than with complete records, reflecting the amount of information recovered by imputing the missing data. In the larger effect example, the conditional distribution of *X*_1_ given *Y* and *X*_2_ obtained from the joint model assumed by JM‐MI is quite different from the one in the data generating mechanism, and hence results are not perfect. Nevertheless, bias is minimal, and the main consequence of the misspecification is an increase in the SEs. In this example, this means there is no information recovery relative to the CR analysis, but model and empirical SEs agree well and hence coverage remains good.

### 3.3 Categorical and ordinal data

In this subsection, we explore various scenarios in which we have at least one categorical variable, with C≥2 categories, among the partially observed variables. The *C* levels of this type of variable can either be ordinal (e.g. items of a Likert scale, “good,” “decent,” “poor”) or unordered categorical (e.g. ethinicity, “Asian,” “black,” “white”).

Goldstein et al. (2009) proposed using a proportional probit model for imputing ordinal data, i.e. one based on a single latent normal with thresholds defining different levels. This is implemented in the standalone software REALCOM (Carpenter et al., 2011), but not in jomo. Therefore, at the end of this subsection, we evaluate imputing ordered categorical data using the unordered latent normal imputation model.

#### 3.3.1 Multinomial logistic regression

Following the pattern of the simulations above, we begin with the categorical variable as the dependent variable in our substantive model, simulating data from the multinomial logistic model as follows:

(10) $$\begin{array}{c} X_{1},X_{2}∼N\left(0,1\right)\\ Z_{2}=\beta_{0,2}+\beta_{1,2}X_{1}+\beta_{2,2}X_{2}\\ Z_{3}=\beta_{0,3}+\beta_{1,3}X_{1}+\beta_{2,3}X_{2}\\ Z_{4}=\beta_{0,4}+\beta_{1,4}X_{1}+\beta_{2,4}X_{2}\\ \left(p_{2},p_{3},p_{4}\right)=\left(\frac{1}{1+\exp(-Z_{2})},\frac{1}{1+\exp(-Z_{3})},\frac{1}{1+\exp(-Z_{4})}\right)\\ Y∼\text{Multinomial}(n=1,1-p_{2}-p_{3}-p_{4},p_{2},p_{3},p_{4}). \end{array}$$

We set $\left(\beta_{0,2},\beta_{1,2},\beta_{2,2}\right)=\left(0,-0.1,0.05\right)$, $\left(\beta_{0,3},\beta_{1,3},\beta_{2,3}\right)=\left(0,-0.05,0\right)$, and $\left(\beta_{0,4},\beta_{1,4},\beta_{2,4}\right)=\left(0,0.05,0.1\right)$, and simulate 1000 datasets of 300 observations, setting 20% of values independently MCAR in *Y* and *X*_2_, and MAR given *Y* in *X*_1_. The substantive model is the multinomial logistic regression model on $X_{1},X_{2}$. As before, we compare complete records, JM‐MI and FCS‐MI.

#### 3.3.2 Results

The results, shown in the top box of Table 3, show latent normal variable approach performs well even with four categories, with all the nine parameter estimates are unbiased, coverage levels achieving the 95% nominal level, mSE's and eSE's similar and always smaller than with CR.

**Table 3:** Simulation results with data generated from (i) (10) (multinomial logit); (ii) (11) (categorical covariate); (iii) (12) (ordinal logit), and (iv) (13) (ordinal covariate)

	β_0_				β_1_				β_2_				β_3_				β_4_			
	Mean	mSE	eSE	Cov	Mean	mSE	eSE	Cov	Mean	mSE	eSE	Cov	Mean	mSE	eSE	Cov	Mean	mSE	eSE	Cov
**Multinomial logit – 2 vs 1**																				
True value	0.00			0.95	−0.10			0.95	0.05			0.95								
Full data	0.00	0.17	0.17	0.94	−0.10	0.17	0.18	0.94	0.05	0.17	0.17	0.95								
Complete records	−0.09	0.22	0.23	0.94	−0.10	0.22	0.23	0.95	0.05	0.22	0.22	0.95								
FCS‐MI	−0.00	0.19	0.19	0.94	−0.11	0.20	0.21	0.93	0.05	0.21	0.21	0.95								
JM‐MI	−0.00	0.19	0.19	0.94	−0.11	0.20	0.21	0.95	0.05	0.21	0.21	0.95								
**3 vs 1**																				
True value	0.00			0.95	−0.05			0.95	0.00			0.95								
Full data	0.00	0.17	0.17	0.95	−0.05	0.17	0.18	0.94	0.00	0.17	0.17	0.95								
Complete records	−0.27	0.23	0.25	0.78	−0.05	0.23	0.24	0.95	0.00	0.23	0.22	0.96								
FCS‐MI	−0.01	0.19	0.20	0.94	−0.05	0.21	0.21	0.95	0.00	0.21	0.21	0.96								
JM‐MI	−0.01	0.19	0.20	0.94	−0.05	0.21	0.21	0.95	0.00	0.21	0.21	0.97								
**4 vs 1**																				
True value	0.00			0.95	0.05			0.95	0.10			0.95								
Full data	−0.00	0.17	0.17	0.96	0.06	0.17	0.17	0.95	0.11	0.17	0.17	0.94								
Complete records	−0.68	0.27	0.28	0.27	0.06	0.27	0.27	0.95	0.11	0.26	0.27	0.96								
FCS‐MI	−0.02	0.19	0.19	0.94	0.05	0.23	0.24	0.95	0.10	0.21	0.21	0.95								
JM‐MI	−0.02	0.19	0.19	0.95	0.05	0.23	0.23	0.95	0.11	0.21	0.21	0.95								
**Categorical covariate**																				
True value	0.10			0.95	0.10			0.95	−0.20			0.95	0.05			0.95	0.10			0.95
Full data	0.10	0.13	0.13	0.94	0.09	0.17	0.17	0.96	−0.20	0.16	0.16	0.94	0.05	0.23	0.23	0.94	0.10	0.06	0.06	0.94
Complete records	−0.09	0.18	0.18	0.80	0.07	0.23	0.23	0.95	−0.19	0.21	0.22	0.95	0.03	0.31	0.31	0.95	0.09	0.08	0.08	0.94
FCS‐MI	0.11	0.17	0.18	0.93	0.08	0.22	0.24	0.93	−0.21	0.21	0.22	0.94	0.04	0.30	0.32	0.93	0.10	0.07	0.08	0.94
JM‐MI	0.10	0.17	0.17	0.94	0.08	0.23	0.23	0.95	−0.20	0.21	0.22	0.94	0.04	0.31	0.31	0.95	0.10	0.07	0.08	0.93
**Ordered logit**																				
True value	0.10			0.95	0.10			0.95	−0.60			0.95	0.00			0.95	0.60			0.95
Full data	0.10	0.11	0.11	0.94	0.10	0.11	0.11	0.96	−0.61	0.12	0.12	0.95	−0.01	0.12	0.11	0.95	0.60	0.12	0.12	0.95
Complete records	0.10	0.15	0.16	0.94	0.09	0.15	0.16	0.93	−0.34	0.16	0.16	0.64	0.34	0.16	0.17	0.44	1.01	0.18	0.19	0.41
FCS‐MI	0.10	0.14	0.14	0.94	0.10	0.13	0.14	0.94	−0.62	0.14	0.14	0.95	−0.00	0.13	0.13	0.94	0.61	0.14	0.14	0.94
JM‐MI	0.10	0.14	0.14	0.94	0.10	0.13	0.14	0.95	−0.62	0.14	0.14	0.95	−0.00	0.13	0.13	0.95	0.61	0.14	0.14	0.94
**Ordinal covariate**																				
True value	0.10			0.95	0.10			0.95	0.20			0.95	0.30			0.95	0.10			0.95
Full data	0.10	0.10	0.11	0.95	0.09	0.17	0.17	0.95	0.20	0.17	0.17	0.93	0.30	0.15	0.14	0.96	0.10	0.06	0.06	0.95
Complete records	−0.04	0.14	0.14	0.79	0.08	0.22	0.22	0.95	0.18	0.22	0.24	0.93	0.26	0.19	0.20	0.95	0.09	0.08	0.08	0.93
FCS‐MI	0.10	0.13	0.14	0.93	0.09	0.21	0.22	0.93	0.20	0.22	0.23	0.94	0.30	0.19	0.19	0.94	0.10	0.07	0.07	0.95
JM‐MI	0.10	0.13	0.14	0.94	0.08	0.22	0.22	0.95	0.20	0.22	0.23	0.94	0.29	0.19	0.19	0.94	0.10	0.07	0.07	0.95

Mean, model SE (mSE), empirical SE (eSE), and coverage level are reported for all model parameters. Data are MAR and we compare complete records (CR) to FCS‐MI and JM‐MI.

#### 3.3.3 Categorical covariate of regression model

Here, we generate a 4‐level categorical *X*_1_:

(11) $$\begin{array}{c} X_{1}∼\mathrm{Multinom}\left(n=1,0.2,0.3,0.4,0.1\right)\;X_{2}∼N\left(0,1\right)\\ Y∼N(\beta_{0}+\beta_{1}I\left[X_{1}=2\right]+\beta_{2}I\left[X_{1}=3\right]+\beta_{3}I\left[X_{1}=4\right]+\beta_{4}X_{2},1), \end{array}$$

where category 1 is used as reference and I[.] is an indicator for the event in brackets. We set $\left(\beta_{0},\beta_{1},\beta_{2},\beta_{3},\beta_{4}\right)=\left(0.1,0.1,-0.2,0.05,0.1\right)$. Once again, we simulate 1000 datasets of 300 observations, make data missing independently on each variables with probability ∼0.2 (MAR given the outcome for *X*_1_), and compare CR, FCS‐MI, and JM‐MI. For FCS‐MI we use multinomial logistic regression for categorical variables and linear regression for normal variables.

#### 3.3.4 Results

**Figure 2:**
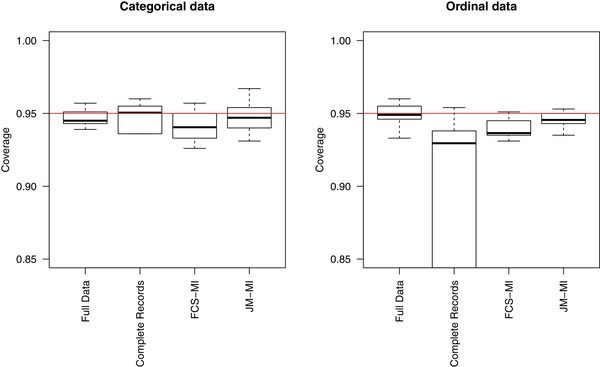
Simulation results for categorical and ordinal data. The left panel summarises the coverage level results across all the parameter estimates and both categorical data scenarios (10) and (11). The right panel gives corresponding results across (12) and (13)

From Table 3 (second box), and the left panel of Figure 2, we see both JM‐MI and FCS‐MI give excellent results.

#### 3.3.5 Ordered logistic regression and ordinal covariate of regression model

We now replace the unordered categorical variable with an ordered categorical variable, and again consider this first as the dependent variable and then as a covariate. The most common regression model for ordinal data is the proportional‐odds ordered logit regression model. Hence, for our first setting with missing ordinal data, we generate data from this model:

(12) $$\begin{array}{c} X_{1},X_{2}∼N\left(0,1\right)\\ Z_{2}=\beta_{0,2}+\beta_{1}X_{1}+\beta_{2}X_{2}\\ Z_{3}=\beta_{0,3}+\beta_{1}X_{1}+\beta_{2}X_{2}\\ Z_{4}=\beta_{0,4}+\beta_{1}X_{1}+\beta_{2}X_{2}\\ \left(p_{2},p_{3},p_{4}\right)=\left(\frac{1}{1+\exp(-Z_{2})},\frac{1}{1+\exp(-Z_{3})},\frac{1}{1+\exp(-Z_{4})}\right)\\ Y∼\text{Multinomial}(n=1,1-p_{2},p_{2}-p_{3},p_{3}-p_{4},p_{4}), \end{array}$$

with $\left(\beta_{0,2},\beta_{0,3},\beta_{0,4},\beta_{1},\beta_{2}\right)=\left(0.1,0.1,-0.6,0,0.6\right)$

For the second scenario, with ordinal covariate, we change the mechanism for generating *X*_1_:

(13) $$\begin{array}{c} Z,X_{2}∼N\left(0,1\right)\\ X_{1}=\left\{\begin{array}{c} 1\;if\;Z<-0.5\\ 2\;if\;-0.5<Z<0\\ 3\;if\;0<Z<0.5\\ 4\;if\;Z>0.5 \end{array}\right.\\ Y∼N(\beta_{0}+\beta_{1}I\left[X_{1}=2\right]+\beta_{2}I\left[X_{1}=3\right]+\beta_{3}I\left[X_{1}=4\right]+\beta_{4}X_{2},\sigma^{2}=1), \end{array}$$

with $\left(\beta_{0},\beta_{1},\beta_{2},\beta_{3},\beta_{4}\right)=\left(0.1,0.1,0.2,0.3,0.1\right)$.

As usual, for both scenarios we generate 1000 simulated datasets of 300 observations, make 20% of the observations on each variable independently missing (MAR for *X*_1_) and compare full data, complete records, JM‐MI and FCS‐MI. With FCS, we use ordered logit to impute the ordinal variable and linear regression to impute the continuous ones, while with JM‐MI we use jomo's generic categorical variable algorithm.

#### 3.3.6 Results

The bottom box of Table 3 and the right panel of Figure 2 give the results. Once again, the latent normal variables JM‐MI algorithm seems to perform well, with negligible information loss when the data are truly ordinal. The results give no reason to suggest that using the generic latent normal categorical algorithm will not work well even if data are truly ordinal. Moreover, using the generic algorithm avoids needing to first check whether, in fact, the ordinal model is appropriate.

### 3.4 Count data

For our final scenario, we consider imputing count data; as before, first when the count is an outcome and second when it is a covariate.

While some promising approaches specifically tailored for the imputation of Poisson data within the JM‐MI framework have been developed (Goldstein & Kounali, 2009), they are not currently implemented in general software. Instead, count variables can either be included in the imputation model as categorical variables, or as continuous. The first option is only viable when the mean of the underlying Poisson distribution is quite low, so that a small number of distinct, low counts are observed. With large counts, the categorical approach becomes unfeasible, and instead we have to treat them as continuous in the JM‐MI framework. It is generally thought that Poisson distributions with mean >20 can be well approximated by a normal distribution. However, as in the Poisson distribution the variance depends from the mean, the variance‐stabilizing square‐root transformation could be helpful. Therefore, we compare the behavior of JM‐MI including our count variable in the model either untransformed or square rooted. Additionally, since the link function for Poisson regression models is the logarithm, we explore the behavior of the log transformation.

As usual, we divide our analysis in two parts, first when the count variable is the dependent variable in the substantive model and second when it is a covariate.

#### 3.4.1 Poisson regression model

We generate data from a Poisson regression model:

(14) $$\begin{array}{c} X_{1},X_{2}∼N\left(0,1\right)\\ \log\left(\lambda \right)=\beta_{0}+\beta_{1}X_{1}+\beta_{2}X_{2}\\ Y∼\mathrm{Poisson}(\lambda ). \end{array}$$

We investigate first a situation in which $\beta_{0}=3$, leading to a quite large average λ∼20, and second one where $\beta_{0}=-0.5$, leading to average λ∼0.7. For the first situation we consider (i) a small ($\beta_{1}=\beta_{2}=0.1$) covariate effect and (ii) a larger effect ($\beta_{1}=\beta_{2}=0.3)$. With the larger covariate effect, for each unit increase in *X*_1_ or *X*_2_, λ increases by 7.

We generate 1000 datasets each of size 300, introduce 20% MCAR data independently in *Y* and *X*_2_, and MAR in *X*_1_, and compare the results of full data and complete records analyses with those after MI. For FCS‐MI, we use predictive mean matching to impute count variables, as Poisson imputation is not implemented in the R package *mice*, although an add‐on package for imputing Poisson data, *countimp* (Kleinke & Reinecke, 2013), has been recently published. For JM‐MI, we compare the results including the count variable in the model either untransformed, square rooted, or log‐transformed. In the scenario with small β_0_, we also treat the count variable as categorical.

#### 3.4.2 Results

**Figure 3:**
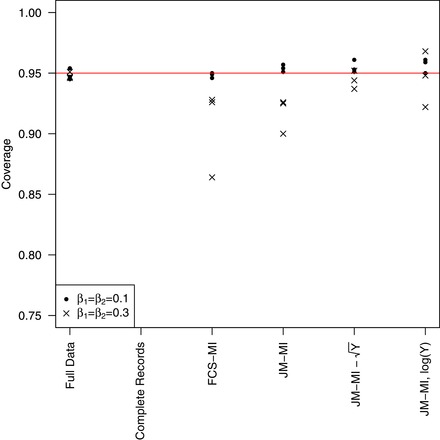
Simulation results for count data: coverage level for the three parameter estimates in the Poisson regression examples with $\beta_{1}=\beta_{2}=0.1$ and $\beta_{1}=\beta_{2}=0.3$

The top three boxes of Table 4 show the results. In the scenarios with larger λ, all JM‐MI methods seem to behave well when the covariate effects are small. However, with larger effects, the variance‐stabilizing transformation leads to better parameter estimates, while—primarily because of small bias in the parameter estimates—the untransformed *Y* method leads to undercoverage for the two slope parameters (Figure 3). Even here, though, JM‐MI outperforms FCS. The log transformation leads to accurate results, although the standard errors get inflated for increasing values of β_1_ and β_2_ (results not shown).

**Table 4:** Count data simulation results, with Poisson dependent data generated from models (14) (boxes 1–3) and Poisson covariate data generated from (15)

	β_0_				β_1_				β_2_			
	Mean	mSE	eSE	Cov	Mean	mSE	eSE	Cov	Mean	mSE	eSE	Cov
**Poisson** – β=(3,0.1,0.1)												
True value	3.00			0.95	0.10			0.95	0.10			0.95
Full data	3.00	0.01	0.01	0.94	0.10	0.01	0.01	0.95	0.10	0.01	0.01	0.95
Complete records	3.06	0.02	0.01	0.06	0.07	0.02	0.02	0.71	0.07	0.02	0.02	0.69
FCS‐MI	3.00	0.02	0.02	0.95	0.10	0.02	0.02	0.95	0.10	0.02	0.02	0.95
JM‐MI, unstransformed Y	3.00	0.02	0.02	0.96	0.10	0.02	0.02	0.95	0.10	0.02	0.02	0.95
JM‐MI, square root of Y	3.00	0.02	0.02	0.96	0.10	0.02	0.02	0.95	0.10	0.02	0.02	0.95
JM‐MI, log(Y)	3.00	0.02	0.02	0.96	0.10	0.02	0.02	0.95	0.10	0.02	0.02	0.96
**Poisson** – β=(3,0.3,0.3)												
True value	3.00			0.95	0.30			0.95	0.30			0.95
Full data	3.00	0.01	0.01	0.95	0.30	0.01	0.01	0.95	0.30	0.01	0.01	0.95
Complete records	3.04	0.02	0.02	0.32	0.27	0.02	0.02	0.62	0.27	0.02	0.02	0.65
FCS‐MI	2.99	0.02	0.02	0.86	0.29	0.02	0.02	0.93	0.30	0.02	0.02	0.93
JM‐MI, unstransformed Y	2.99	0.02	0.02	0.90	0.29	0.02	0.02	0.92	0.31	0.02	0.02	0.93
JM‐MI, square root of Y	3.00	0.02	0.02	0.94	0.30	0.02	0.02	0.95	0.30	0.02	0.02	0.94
JM‐MI, log(Y)	3.01	0.02	0.02	0.92	0.30	0.02	0.02	0.97	0.29	0.02	0.02	0.95
**Poisson** – β=(−0.5,0.1,0.1)												
True value	−0.50			0.95	0.10			0.95	0.10			0.95
Full data	−0.51	0.08	0.08	0.95	0.10	0.07	0.08	0.95	0.10	0.07	0.07	0.96
Complete records	−0.38	0.10	0.10	0.73	0.09	0.10	0.10	0.94	0.09	0.10	0.09	0.96
FCS‐MI	−0.52	0.09	0.09	0.95	0.10	0.09	0.09	0.95	0.10	0.09	0.09	0.96
JM‐MI, unstransformed Y	−0.48	0.08	0.08	0.94	0.10	0.09	0.09	0.95	0.09	0.09	0.08	0.95
JM‐MI, square root of Y	−0.51	0.09	0.09	0.95	0.10	0.09	0.09	0.95	0.09	0.09	0.08	0.96
JM‐MI, log(Y)	−0.49	0.10	0.09	0.97	0.11	0.11	0.10	0.97	0.10	0.11	0.09	0.98
JM‐MI, categorical Y	−0.52	0.09	0.09	0.95	0.10	0.09	0.09	0.95	0.09	0.09	0.09	0.95
**Count covariate**												
True value	0.30			0.95	0.30			0.95	0.30			0.95
Full data	0.31	0.12	0.12	0.95	0.30	0.03	0.04	0.94	0.30	0.06	0.06	0.95
Complete records	0.24	0.15	0.16	0.92	0.27	0.05	0.05	0.87	0.27	0.08	0.08	0.94
FCS‐MI	0.33	0.15	0.16	0.93	0.29	0.04	0.05	0.93	0.30	0.07	0.07	0.95
JM‐MI, unstransformed Y	0.32	0.15	0.15	0.94	0.30	0.04	0.05	0.94	0.30	0.07	0.07	0.95
JM‐MI, square root of Y	0.39	0.15	0.14	0.91	0.27	0.04	0.04	0.89	0.30	0.08	0.07	0.95
JM‐MI, log(Y)	0.98	0.14	0.10	0.00	0.06	0.04	0.02	0.00	0.29	0.08	0.07	0.96

Mean, model SE (mSE), empirical SE (eSE), and coverage level for each parameter. Data are MAR (see text) and we compare complete records (CR) to both FCS and JM imputation. For JM imputation we compare including Y either untransformed, square rooted, log‐transformed or as a categorical variable.

With small λ, the method including *Y* as categorical in the imputation model leads to similarly good results.

In summary, when including a count outcome variable in the imputation model with JM‐MI, if λ is small the categorical approach is viable and effective, while with larger values of λ, *Y* can be included in the model as continuous, possibly after a log or square‐root transformation.

#### 3.4.3 Count covariate of a regression model

Although less common, a count variable may be included in a regression model as a covariate. Here, we explore this by simulating 1000 datasets of size 300 from the following distribution:

(15) $$\begin{array}{c} X_{1}∼Poisson\left(3\right)\;X_{2}∼N\left(0,1\right)\\ Y∼N(\beta_{0}+\beta_{1}X_{1}+\beta_{2}X_{2},1), \end{array}$$

with $\left(\beta_{0},\beta_{1},\beta_{2}\right)=\left(0.3,0.3,0.3\right)$. As in the previous example, we compare three different ways of performing JM‐MI, always including *X*_1_ as continuous, but either untransformed, square rooted, or log‐transformed.

#### 3.4.4 Results

When the partially observed count variable is included as a covariate in the substantive analysis model, results of these simulations clearly show that the log transformation is a poor option. This is because it is inconsistent with the linear relationship in (15). This is also the reason why the variance‐stabilizing square‐root transformation struggles. Instead, including it in the imputation model untransformed is the best option, as it does not alter the linear association of the variable with the outcome (see e.g. Lee & Carlin, 2017).

## 4 A REAL DATA EXAMPLE: THE GERMAN BREAST CANCER STUDY GROUP

Here, we evaluate the latent normal JM‐MI approach for the imputation of real, rather than simulated, data, again comparing the results with those obtained using FCS‐MI.

We use data from a prospective study on node‐positive breast cancer patients of the German Breast Cancer Study Group (Sauerbrei, Royston, Bojar, Schmoor, & Schumacher, 1999; Schumacher et al., 1994). The study was a Comprehensive Cohort Study, where patients satisfying eligibility criteria were asked whether they consented to be randomized; otherwise, physicians chose their preferred treatment.

Table 5 gives a summary of the baseline characteristics for the 686 patients included in the study. Suppose we are interested in the effect of hormone therapy on survival. For this purpose, we want to fit a Cox proportional hazards model with hormone therapy as the main exposure, adjusting for age, tumor size and grade, number of active nodes and menopausal state. Following (Sauerbrei et al., 1999), the number of nodes is exponentially transformed to reflect medical knowledge about the effect on survival.

**Table 5:** Baseline characteristics of the German Breast Cancer Group data

Variable	Overall
*n*	686
nodes (median [IQR])	3 [1, 7]
grade (1	81 (11.8)
2	444 (64.7)
3	161 (23.5)
meno = premenopausal (%)	290 (42.3)
age (mean (sd))	53.05 (10.12)
hormon = no tamoxifen (%)	440 (64.1)
size (mean (sd))	29.33 (14.30)

In order to evaluate the imputation methods, we sample with replacement from the original data 1000 datasets with the same sample size, and in each of these we introduce missing data in three variables, that we later impute with either FCS‐MI or JM‐MI:

1. Tumor grade: this is a 3‐level categorical variable, and we make ∼20% missing with an MAR mechanism dependent on the outcome (survival time). Being an ordered variable, we impute it with an ordinal regression model within FCS‐MI while in JM‐MI we handle it with two latent normal variables. Given the results of the simulation section, we expect this choice not to lead to excessive loss of efficiency compared to a model where the order between categories is acknowledged;
2. Menopausal state: this variable is binary, and we make ∼20% observations missing with an MAR mechanism dependent on age at recruitment; we include it in the imputation model for JM‐MI with a latent normal variable, while for FCS‐MI, we use a simple logistic regression model;
3. Number of active nodes: this is a count variable, and we introduce 20% missing values with an MCAR mechanism. We use PMM for FCS‐MI, and, given the results of the simulation section, we do not further transform the variable before including it in the JM‐MI imputation model.

Given that the analysis model is a Cox regression model, we follow the suggestion in White and Royston (2009) and include the Nelson‐Aalen estimator and the event indicator in the imputation model. For each sample, we create 20 imputed datasets.

**Figure 4:**
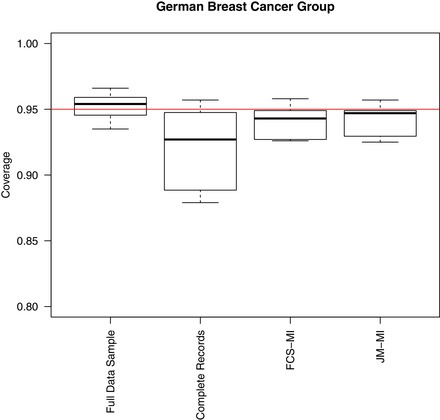
Results of the resampling study with the German Breast Cancer Group Data: boxplot of coverage levels for parameter estimates in the survival model

Figure 4 compares coverage levels for the set of fixed effect parameter estimates from full data analysis, and handling missing data with either complete records, FCS‐MI or JM‐MI. Coverage levels for both imputation methods are close to 95%, although on average slightly below the nominal level because of small biases in some parameter estimates. Hence, a comparison between the two imputation strategies is consistent with the results from the simulation studies, and confirms that the two methods perform very similarly even with real data and a larger number of variables.

## 5 DISCUSSION

In this paper, we reported the results of a comprehensive series of simulation studies to investigate the validity of the latent normal variables approach to include binary, categorical, ordinal, and count variables in the Joint Modelling imputation framework. We explored both cases where such data were included as outcomes or covariates in our substantive analysis models. Our results show that, provided we choose an appropriate imputation model—at least approximately congenial with the analysis model of interest—this approach gives valid results across a range of scenarios, and sometimes outperforms FCS.

Starting with binary and categorical outcome data, the results were uniformly good. With categorical covariate data, in most situations results were very good, with only a single scenario where, with very strong covariate effects, inference after JM‐MI led to slight overestimation of the standard errors. The underlying cause of this is that the data generating mechanism (from the general location model) was not fully compatible with the latent normal model. While analysts need to be aware this may be an issue, the parameters used in the scenario were purposely quite extreme, and hence we believe that in the large majority of settings this is of negligible consequence in practice.

Turning to ordinal data, our results showed that the general latent normal variables algorithm for dealing with categorical variables works very well for ordinal data, with negligible loss of efficiency. Thus automatic use of the general categorical model is a sensible approach in applications, as it also avoids the need to first establish whether the simpler proportional probit/logit model is plausible.

Lastly, we considered count data. When the count is the dependent variable and the mean small, best results were achieved by treating it as categorical. When the mean is larger, best results were achieved with a log transformation (consistent with the log‐linear relationship in the substantive model) although square root and untransformed approaches also performed reasonably. However, when the count is a covariate, the log or square root transformation should not be used, because they are both markedly inconsistent with the substantive model. Instead, including the count variable untransformed was the best approach. Note that, across all the simulations, we did not round imputed values of count outcome variables, but only truncated them at 0 to avoid negative counts.

We compared JM‐MI with FCS‐MI in each scenario, finding that the two methods performed similarly in most situations, although as detailed in the results, in some settings FCS‐MI was slightly worse than JM‐MI and vice versa. Therefore, in practice we believe that users can decide to use either of the two methods interchangeably. At least compared to the current implementation of *jomo*, FCS‐MI potentially has an advantage compared to JM‐MI in terms of computational time with large datasets, particularly with a large number of categorical variables. The big advantage of JM‐MI is that it involves an explicit formulation of the joint imputation model (so it avoids issues of compatibility between the univariate models) and it extends naturally to the multilevel setting.

Other papers have compared JM‐MI with FCS‐MI with noncontinuous data in recent years, but most of these papers used different strategies for including categorical variables; for example, Lee and Mitra (2016) used sequential generalized linear models, while (Wu, Jia, & Enders, 2015) used an ordered version of the latent normal model, which matches our model only for the imputation of binary variables. Other simulation studies made use of our latent normal approach to compare FCS‐MI and JM‐MI in specific settings, e.g. (Kalaycioglu, Copas, King, & Omar, 2016) focused on repeated‐measurements observational studies, and (Audiger et al., 2018) on multilevel analyses. Finally, Zhang, Boscardin, and Belin (2008) evaluated the method of latent normal variables when performing a fully Bayesian analysis.

All JM‐MI analyses have been performed with R package *jomo*, freely available on CRAN. The package also allows for the imputation of clustered and two‐level data, handling missing values of all types at both levels, and allowing for cluster‐specific covariance matrices. As already mentioned, recently, using this package, the latent normal JM‐MI approach has been compared in the multilevel MI settings to one‐stage and two‐stage FCS (Audiger et al., 2018) in the context of individual patient data meta‐analysis, giving good results even when the data generating mechanism is inconsistent with the joint model, and giving the least biased results when imputing binary covariates of a substantive linear‐mixed model.

None of the substantive models used in this paper included interactions or nonlinear relationships. When some of the partially observed variables are included in the substantive model of interest as covariates with an interaction or a nonlinearity, imputation that does not appropriately respect this is invalid. A possible solution is to make use of substantive model compatible imputation: with JM‐MI, the basic idea is to split the imputation model in two parts, defining a model for the covariates and a conditional model for the outcome given the covariates (Goldstein, Carpenter, & Browne, 2014). Since the whole joint model is not of a known form, we need to extend the Gibbs sampler with either a Metropolis–Hastings step or rejection sampling. We recently included in jomo some features supporting substantive model compatible imputation. This method could also be extended to improve the imputation of count outcome variables, allowing for imputation compatible with a Poisson regression model. Similarly, it could be used for imputing compatibly with any survival analysis model (Bartlett, Seaman, White, & Carpenter, 2015; Keogh & Morris, 2018).

In summary, the results of our comprehensive set of simulation studies show that researchers can use JM‐MI, implemented in jomo, for imputation of missing binary, categorical, ordinal, and count data (as well as continuous data) with confidence.

## CONFLICT OF INTEREST

The authors have declared no conflict of interest.

## Supporting information

Supplementary Material
